# Can Sequence Phylogenies Safely Infer the Origin of the Global Virome?

**DOI:** 10.1128/mBio.00289-19

**Published:** 2019-04-16

**Authors:** Edward C. Holmes, Sebastián Duchêne

**Affiliations:** a Marie Bashir Institute for Infectious Diseases and Biosecurity, The University of Sydney, Sydney, NSW, Australia; b Charles Perkins Centre, School of Life and Environmental Sciences, The University of Sydney, Sydney, NSW, Australia; c Sydney Medical School, The University of Sydney, Sydney, NSW, Australia; d Department of Microbiology and Immunology, Peter Doherty Institute for Infection and Immunity, University of Melbourne, Melbourne, VIC, Australia; Columbia University College of Physicians & Surgeons

**Keywords:** evolution, phylogeny, RNA polymerase, virus

## LETTER

Resolving the origins of RNA viruses is one of the most important questions in viral evolution, but research on this question has been hampered by a lack of primary sequence similarity among the most divergent groups of RNA viruses (1). In a recent paper, Wolf et al. (2) seemingly overcame these problems and presented a comprehensive picture of the origins and deep evolutionary relationships among divergent RNA viruses. While many of the ideas presented in this study (2) were illuminating, we contend that they cannot be supported by the phylogenetic analysis performed, which is still based on sequences that often share no recognizable similarity and cannot be safely aligned.

Central to the study of Wolf et al. (2) is the quality of their sequence alignment of the most conserved RNA-dependent RNA polymerase (RdRp) protein. To obtain this, Wolf et al. (2) followed an iterative procedure, resulting in a main alignment comprising 4,627 taxa with a length of 12,220 amino acids. However, even a cursory inspection of this alignment indicates that it is highly unlikely to be accurate among the most distantly related RNA viruses. For example, (i) every site in the alignment contains at least one gap, including the canonical GDD motif, and there are no contiguous stretches of clearly aligned sequence across all viruses. (ii) Only 3.6% (441 residues) of the alignment remains after sites that harbor a majority (>50%) of gaps are removed. (iii) The pairwise identity between the aligned sequences is often less than the 5% expected by chance alone (mean value of 7.7% across the alignment) and was as low as 1%, with a mean pairwise distance of 0.93 (from a maximum of 1) substitutions per site. (iv) A total of 812 sites contain all 20 amino acids, and (v) 95.9% of sequences failed a χ^2^ test of compositional heterogeneity in IQ-TREE (3). (vi) Only six sites can be safely aligned according to Gblocks (4), whereas TrimAl (5) could not align any sites, with both programs employing their least stringent settings. Points iii to v imply that even sophisticated substitution models cannot reliably estimate evolutionary divergence in these data (6), and point vi is particularly important for phylogenetic inference because the inclusion of poorly aligned regions results in biased tree estimates, with high bootstrap support for the incorrect topology (4). While clusters of clearly related sequences are present in these data, the deepest parts of the phylogeny reflect sequences that are so divergent in sequence that any attempt to depict their relationship, including through bootstrap analysis, is meaningless.

Despite the presence of a number of putative sequence motifs that we agree are indicative of common ancestry, the sequence alignment presented by Wolf et al. (2) is not sufficiently robust for a comprehensive phylogenetic analysis or to draw conclusions about the earliest moments of RNA virus evolution. We urge that caution be exercised in all studies that utilize sequences as divergent as those analyzed here, as phylogenies are meaningful only when they are estimated in the case of clear primary sequence similarity. Unfortunately, this is unlikely ever to be realistic in the case of RNA viruses.
